# Development of a High-Sensitivity Humidity Sensor Using Fiber Bragg Grating Coated with LiCl@UIO-66-Doped Hydrogel

**DOI:** 10.3390/ma18245587

**Published:** 2025-12-12

**Authors:** Binxiaojun Liu, Zelin Gao, Runqi Yao, Liyun Ding, Xusheng Xia

**Affiliations:** 1 The National Engineering Research Center of Fiber Optic Sensing Technology and Network, Wuhan University of Technology, Wuhan 430070, China; 2 School of Physical Science and Technology, Lanzhou University, Lanzhou 730000, China

**Keywords:** hydrogel humidity sensor, Fiber Bragg grating (FBG), LiCl@UIO-66, PNIPAM composite hydrogel, strain transfer

## Abstract

Humidity monitoring is essential in industrial and scientific scenarios, yet remains challenging for compact EMI (electromagnetic interference)-immune sensors with high sensitivity and robust stability. A novel fiber Bragg grating (FBG) humidity sensor was developed, which incorporated LiCl@UIO-66 microfillers within a poly(N-isopropylacrylamide) (PNIPAM) hydrogel matrix. Structural characterization using X-ray diffraction (XRD), scanning electron microscopy (SEM), energy-dispersive X-ray spectroscopy (EDS), and Fourier-transform infrared (FTIR) spectroscopy confirms that LiCl is confined or nanodispersed within intact UIO-66, and that interfacial ion–dipole/hydrogen-bonding exists between the composite and water. Systematic variation in coating time (30–720 min) reveals monotonic growth of the total wavelength shift with diminishing returns. A coating time of 4 h was found to yield a wavelength shift of approximately 0.38–0.40 nm, representing about 82% of the maximum shift observed at 12 h, while maintaining good quasi-linearity and favorable kinetics. Calibration demonstrates sensitivities of 6.7 pm/%RH for LiCl@UIO-66_33 and 10.6 pm/%RH for LiCl@UIO-66_51 over ~0–95%RH. Stepwise tests show response times t90 of ≈14 min for both composites, versus ≈30 min for UIO-66 and ≈55 min for neat PNIPAM. Long-term measurements on the 51 wt.% device are stable over the first ~20 days, with only slow drift thereafter, and repeated humidity cycling is reversible. The wavelength decreases monotonically during drying while settling time increases toward low RH. The synergy of hydrogel–MOF–salt underpins high sensitivity, accelerated transport, and practical stability, offering a scalable route to high-performance optical humidity sensing.

## 1. Introduction

Humidity is a critical environmental parameter in industrial and scientific contexts, including food processing, agriculture, meteorology, civil engineering, and biomedicine—where precise measurement and control are indispensable for quality assurance, safety, and comfort [1,2,3]. Examples range from moisture management during food preservation and pharmaceutical manufacturing to conservation of artworks [4,5]. In civil infrastructure, humidity monitoring offers insight into concrete health and mitigates potential risks [6,7], while human thermal comfort and health are tightly coupled to ambient humidity. These demands motivate sensing technologies that are accurate, robust, and deployable in diverse environments.

Conventional electrical humidity sensors (capacitive or resistive) are widely used but can be susceptible to electromagnetic interference and often require protective electronics; thermal-conductivity devices typically need more complex circuitry [8,9]. By contrast, optical fiber-based sensors combine in situ and remote operation with intrinsic immunity to electromagnetic fields, compact size, low mass, and explosion safety, making them attractive for harsh or constrained settings [10,11]. Among optical approaches, fiber Bragg grating (FBG)-based sensors are particularly compelling because their wavelength-encoded signal is resilient to intensity fluctuations and amenable to wavelength-division multiplexing for distributed measurements [12,13,14].

Optical fiber gratings—principally fiber Bragg gratings (FBGs) and long-period gratings (LPGs)—are widely used transducers in fiber-optic sensing [15,16,17]. They are permanently inscribed in silica fibers and inherit the well-known advantages of optical fiber sensors, including electromagnetic immunity, small size, remote interrogation, and straightforward multiplexing. FBG humidity sensing is commonly realized by coating the grating with a hygroscopic layer that swells or contracts with relative humidity (RH). The volume change induces axial strain in the FBG and produces a Bragg wavelength shift that can be demodulated to quantify RH [18]. Consequently, sensor performance is governed by the coating’s properties, such as sensitivity, linearity, hysteresis, response/recovery time, stability, and adhesion [19]. A variety of materials have been explored, including polyimide (PI) [20,21], PMMA [22], PVA [23], graphene oxide (GO) [24,25], gelatin [26], and hydrogels [27]. Hydrogels are three-dimensional polymer networks with high water uptake that preserve structural integrity; they have been widely used in hygrometers, contact lenses, biosensing, and drug delivery [28,29,30]. When integrated with FBGs, hydrogels can translate moisture sorption into large and reversible strain, offering a promising platform for high-performance RH sensing [31,32]. Recent progress in functional hydrogel design shows that incorporating nanoscale fillers or engineered microstructures can markedly enhance mechanical robustness and transduction performance [33]. In addition, optimized polymer architectures and hybrid organic–inorganic networks have been shown to improve stability and water-management capability in biomedical hydrogels [34]. These advances highlight the importance of rational hydrogel design for achieving high humidity sensitivity, fast response, and long-term stability in FBG-based humidity sensors.

Prior studies have demonstrated the feasibility of hydrogel-coated optical fibers. For example, a chitosan-coated tilted FBG (TFBG) enabled simultaneous temperature–RH discrimination by exploiting an RH-insensitive Bragg peak with a temperature sensitivity of 8.7 ± 0.1 pm/°C, together with a cladding-mode spectral minimum exhibiting sensitivities of 8.0 ± 0.1 pm/°C (temperature) and 0.88 ± 0.06 pm/%RH (humidity) [35]. In parallel, agarose hydrogel-coated photonic crystal fiber interferometers have also been explored. Despite these advances, further improvements in RH sensitivity, linearity over a wide humidity range, and response speed are still needed while maintaining long-term stability.

In this work, we address these needs by employing a hygroscopic hydrogel coating in which LiCl@UIO-66 is dispersed within a poly(N-isopropylacrylamide) (PNIPAM) matrix. Here, UIO-66 (University of Oslo-66) is a zirconium-based metal–organic framework constructed from Zr_6_O_4_(OH)_4_ clusters and terephthalate (BDC) linkers, providing microporous confinement and rapid transport pathways for LiCl. In this composite, LiCl contributes strong hygroscopicity and high hydration numbers, while the PNIPAM network converts water uptake into axial strain on the FBG. The result is a coating that couples large hygroscopic expansion with efficient strain transfer and robust adhesion. Systematic evaluation of the sensors demonstrates high sensitivity, with a calibration slope up to 10.6 pm/%RH, together with excellent linearity, repeatability, and stability. Response times on the order of minutes are achieved, and optimization of coating thickness (via deposition time) identifies a practical operating point that balances sensitivity with kinetics. These findings highlight the hydrogel–MOF–salt architecture as a scalable route to high-performance, EMI-immune optical humidity sensing suitable for multiplexed FBG interrogation in demanding environments.

## 2. Theory and Experiments

### 2.1. Theory

Fiber Bragg gratings in bare form respond mainly to mechanical strain and temperature; selectivity to relative humidity (RH) is obtained by functionalizing the fiber with a hygroscopic coating that transduces moisture uptake into an optical change while maintaining strong adhesion to the glass surface [36].

An FBG reflects a narrowband spectrum centered at the Bragg wavelength:

(1) $$\lambda_{B}=2\eta_{eff}$$

where n_eff_ is the effective refractive index of the guided core mode and Λ\LambdaΛ is the grating period. Small perturbations in strain ε/varepsilonε and temperature ΔT shift λB according to

(2) $${\Delta \lambda }_{B}/\lambda_{B}=\left(1-p_{e}\right)\cdot \varepsilon +\left[\left(1-p_{e}\right)\cdot \alpha_{\Lambda }+\alpha_{n}\right]\cdot \Delta T$$

where p_e_ is the effective photoelastic coefficient of the fiber, α is the thermal expansion coefficient of silica, and ξ is the thermo-optic coefficient. In an RH sensor, a hygroscopic overlayer swells or shrinks with ambient humidity and imposes an axial strain on the underlying FBG; the induced ε/varepsilonε gives a proportional Δλ_B_, enabling quantitative RH readout. For LPGs, RH can modulate both coating-induced strain and the surrounding refractive index, altering core–cladding mode coupling and the transmitted resonance wavelengths.

Two interrogation geometries are typically used. In reflection mode (standard for FBGs), broadband light is launched into the fiber, and the back-reflected spectrum is routed by a three-port optical circulator to a spectrometer/OSA, isolating it from the source. In transmission mode (common for LPGs), light passes directly to a detector/OSA, sampling interactions between the guided modes and the sensitive coating along the grating length. FBGs can also be operated in reflection after a distal mirror is added if needed, whereas LPG-based reflection requires a reflective termination.

The functional coating is central to RH transduction. Practical layers combine the following: (i) high, reversible water uptake; (ii) fast sorption/desorption kinetics; (iii) minimal hysteresis and aging; (iv) robust adhesion to silica. Reported materials include polymers such as polyimide (PI), PMMA, PVA, PEG, and agar; carbon-based nanomaterials such as graphene oxide (GO) and carbon nanotubes; metal-oxide or silica nanoparticles (e.g., TiO_2_, SiO_2_); and hydrogels. Among these, hydrogels—three-dimensional polymer networks that absorb large amounts of water while retaining structural integrity—are particularly attractive because their substantial volumetric swelling produces a strong mechanical coupling to the grating, yielding large and often linear wavelength shifts. Accordingly, by engineering the coating composition, microstructure, and thickness, an OFG can be rendered selectively sensitive to humidity while preserving the inherent advantages of fiber-optic sensing.

### 2.2. Materials and Methods

#### 2.2.1. Reagents

ZrCl_4_ (≥99.5%, Macklin, Shanghai, China), terephthalic acid (H_2_BDC, ≥99%, Macklin, Shanghai, China), N-isopropylacrylamide (NIPAM, 99%, Macklin, Shanghai, China), N,N′-methylenebisacrylamide (BIS, 99%, Macklin, Shanghai, China), Irgacure 2959 (≥98%, Macklin, Shanghai, China), 3-(trimethoxysilyl)propyl methacrylate (TMSPMA, 98%, Macklin, Shanghai, China), and all solvents including DMF, ethanol, methanol, acetone, and glacial acetic acid were also obtained from Macklin (Shanghai, China).

#### 2.2.2. Synthesis of UIO-66

UIO-66 was synthesized via a solvothermal method. First, 0.70 g ZrCl_4_ and 0.49 g H_2_BDC were dissolved in 50 mL DMF, followed by the addition of 5 mL HAc as a modulator. The mixture was sonicated for 10 min until clear, transferred to a PTFE-lined autoclave, and heated at 120 °C for 24 h. After reaction, the white precipitate was collected by centrifugation and washed three times with DMF and three times with methanol to remove residual solvent and ligands. The solid was then vacuum-dried at 120 °C for 12 h to obtain activated UIO-66. The product was a white powder; XRD confirmed good crystallinity, and the BET specific surface area was approximately 1000–1200 m^2^·g^−1^.

#### 2.2.3. Preparation of LiCl@UIO-66

LiCl was introduced by solution impregnation with vacuum assistance. Activated UIO-66 was weighed according to the target LiCl loadings of 33 wt% and 51 wt%. For example, with 0.50 g UIO-66, ~0.246 g LiCl is required for 33 wt% and ~0.521 g LiCl for 51 wt%. UIO-66 was dispersed in a 6.0 M LiCl aqueous solution, subjected to three vacuum–nitrogen cycles to promote pore infiltration, and stirred at room temperature for 12 h. The solid was then collected by centrifugation and briefly rinsed with a small amount of acetone to remove surface-adhered salt crystals. After vacuum drying at 80 °C for 6–8 h, LiCl@UIO-66 was obtained. Mass gain was used to estimate the actual LiCl loading, and the crystallinity and elemental distribution were verified by XRD, XPS, and SEM.

#### 2.2.4. Preparation of LiCl@UIO-66/PNIPAM Composite Hydrogel

PNIPAM was selected as the hydrogel matrix and generated in situ through free-radical photopolymerization of the monomer N-isopropylacrylamide (NIPAM, M_w = 113.16 g·mol^−1^, 99% purity) and the crosslinker N,N′-methylenebisacrylamide (BIS, M_w = 154.17 g·mol^−1^, 99% purity). Because PNIPAM forms a covalently crosslinked three-dimensional network, a conventional molecular weight is not applicable; its structure is instead determined by the monomer-to-crosslinker ratio (≈2 mol% BIS in this work).

For a 10 mL prepolymer solution, the formulation consisted of NIPAM (1.00 g), BIS (30 mg), LiCl@UIO-66 (0.10–0.20 g, corresponding to 10–20 wt% relative to NIPAM), and Irgacure 2959 (5 mg) dissolved in a water/ethanol (95:5, *v*/*v*) solvent mixture. LiCl@UIO-66 was first ultrasonically dispersed in 5 mL of the solvent for 15 min, after which NIPAM and BIS were added sequentially until fully dissolved. The photoinitiator was then introduced, and the total volume was adjusted to 10 mL. The resulting mixture was purged with nitrogen for 10 min to remove dissolved oxygen prior to coating. When necessary, a trace amount of poly(vinylpyrrolidone) (PVP) was added to enhance dispersion stability.

#### 2.2.5. FBG Surface Functionalization and in Situ Coating

To enhance adhesion between the coating and the fiber, the FBG surface was pretreated. After sequential rinses with ethanol and water, the fiber could optionally be exposed to O_2_ plasma for 2–3 min to increase surface hydroxyl groups. The fiber was then immersed in 2 vol% TMSPMA in ethanol containing 1 vol% water (pH ≈ 4.5) for 30 min and cured at 110 °C for 10 min. The silanized FBG was dipped into the prepolymer solution and withdrawn at 1–2 mm·s^−1^ to form a coating; multiple cycles were performed if a thicker film was required. The coating was photopolymerized under 365 nm UV irradiation (10 mW·cm^−2^) for 10–15 min. The cured fiber was quickly rinsed with water/ethanol (95:5) to remove residual monomers and dried at 40 °C under vacuum for 1 h. Finally, the samples were conditioned at low humidity (RH ≤ 20%) for 12 h prior to humidity-sensing measurements.

### 2.3. Characterization

The characterization procedures were conducted following commonly used methods reported in previous studies [37,38,39,40,41]. The crystal structures of UIO-66 and LiCl@UIO-66 were examined using a Bruker D8 Advance X-ray diffractometer (Bruker Corporation, Billerica, MA, USA). Powder samples were gently ground in an agate mortar and evenly spread onto a low-background sample holder without pressing to avoid inducing texture or preferred orientation.

Fourier-transform infrared (FTIR) spectra were collected using a Nicolet 6700 FT-IR spectrometer (Thermo Fisher Scientific, Waltham, MA, USA) in the range 400–4000 cm^−1^. For FTIR measurements, approximately 1–2 mg of sample was mixed with ~200 mg of dry KBr, finely ground, and pressed into transparent pellets under vacuum.

Morphologies of UIO-66, LiCl@UIO-66, and the freeze-dried hydrogel were characterized using a JSM-IT300 field-emission scanning electron microscope (SEM) (JEOL Ltd., Tokyo, Japan). Powder samples were dispersed in ethanol, drop-cast onto silicon wafers, dried at 60 °C, and sputter-coated with a thin Au layer (~5 nm). For the composite hydrogel, the coating layer was carefully detached from the fiber, frozen, and freeze-dried to preserve the porous structure before being mounted and sputter-coated.

Chemical composition and elemental distributions of LiCl@UIO-66 were analyzed using an X-MaxN20 energy-dispersive X-ray spectroscopy (EDS) system (Oxford Instruments, Oxford, UK). Elemental mapping was conducted on the same sputter-coated specimens used for SEM imaging.

### 2.4. Reflection-Mode FBG Interrogation and Humidity Control

Humidity measurements were performed on a reflection-mode FBG interrogation platform comprising a broad source, a 3-port optical circulator (1550 nm window), a sealed test chamber housing the coated FBG (commercial, Shenzhen, China; 1550 nm window) and a reference thermo-hygrometer (commercial, Shenzhen, China), and a spectrometer/optical spectrum analyzer (OSA) (commercial, Shenzhen, China). Broadband light enters port-1, is directed to the in-chamber FBG via port-2, and the reflected Bragg spectrum returns through the port to the OSA for real-time tracking of λB. To suppress parasitic reflections, all fiber connections use FC/APC connectors.

**Figure 1:**
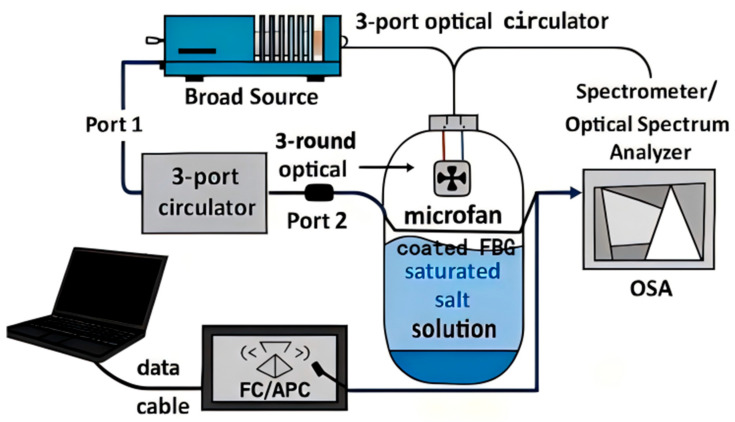
Schematic of the reflection-mode FBG humidity-sensing platform.

Within the chamber, a shallow open dish containing a saturated salt solution establishes a stable relative humidity (RH). The FBG sensing segment is held suspended above the liquid surface with a gap ≥2 cm, while a low-speed micro-fan gently mixes (commercial, Shenzhen, China) the headspace to minimize RH gradients. Temperature drift is monitored and compensated using a PRT probe (Omega Engineering Inc., Norwalk, CT, USA) (or an uncoated reference FBG (commercial, Shenzhen, China)) placed near the sensor. The OSA records the λB–time trace with a spectral resolution ≤0.02 nm; at each RH setpoint, once steady state is reached, the reported value is the average over a 30–60 s window. The overall layout is shown schematically in Figure 1.

RH control follows the saturated-salt method at 25 ± 0.5 °C. For each setpoint, excess solid salt is mixed with a small amount of deionized water to form a slurry-like saturated solution, which is then placed in the chamber’s shallow dish; after natural equilibration, the headspace RH stabilizes at a salt-specific value. The approximate RH setpoints produced by common saturated salts at 25 °C are summarized in Table 1.

## 3. Results and Discussion

### 3.1. Structural and Morphological Characterization

**Figure 2:**
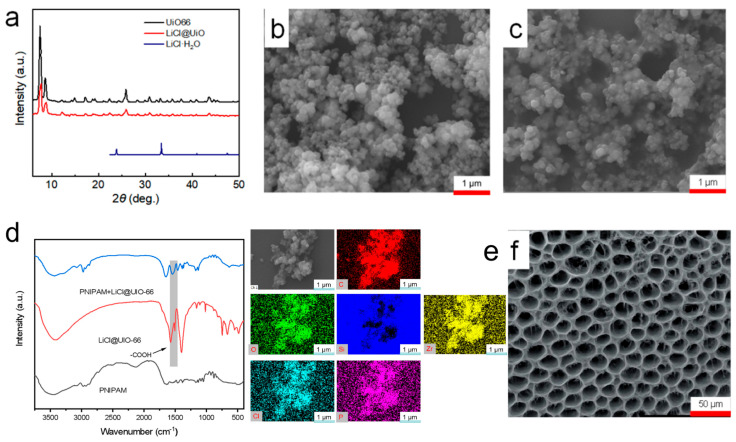
Structural and chemical characterization of UIO-66, LiCl@UIO-66, and the LiCl@UIO-66/PNIPAM hydrogel. (**a**) Powder XRD patterns of UIO-66, LiCl@UIO-66, and reference LiCl·H_2_O. (**b**,**c**) SEM images of pristine UIO-66 and LiCl@UIO-66, respectively (scale bars 1 μm). (**d**) FTIR spectra of PNIPAM, LiCl@UIO-66, and the composite film; UIO-66. (**e**) Representative SEM–EDS elemental maps (Zr, O, C, and Cl) showing uniform Cl distribution; Li is not mapped due to the limited EDS sensitivity. (**f**) SEM image of the composite hydrogel exhibiting a honeycomb-like interconnected macroporous network (scale bar 50 μm).

From the XRD patterns (Figure 2a), the principal reflections of UIO-66 remain at essentially the same 2θ positions before and after loading; the resolvable peak shift is <0.2°. Relative to the unloaded sample, LiCl@UIO-66 exhibits an ≈20–30% overall decrease in peak intensity together with slight peak broadening, while the characteristic reflections of the reference LiCl·H_2_O phase are absent. These data indicate that the crystallographic framework is preserved. The attenuated intensity and broader peaks are more plausibly attributed to in-pore guest–induced scattering/absorption and changes in microcrystal size/microstrain, rather than framework damage. The lack of LiCl·H_2_O peaks further suggests that LiCl resides confined within the pores or as nanodispersed species inside UIO-66, rather than crystallizing on the particle surface.

SEM images (Figure 2b,c) show that UIO-66 consists of submicrometer, roughly equiaxed particles with a characteristic size of hundreds of nanometers. After LiCl loading, the particle shape and degree of agglomeration are essentially unchanged, and no plate-like or prismatic salt crystallites are observed on the surface. Together with the XRD results, this implies that the vacuum impregnation plus rapid organic rinse effectively suppresses surface crystallization and drives LiCl to preferentially enter and remain in the MOF pores. The strong hygroscopicity of LiCl may induce slight particle cementation, but its effect on the apparent size is limited.

Elemental mapping by SEM–EDS (Figure 2e) further reveals that the signals of Zr, O, and C delineate the MOF particle contours, while Cl is uniformly distributed and spatially overlaps with Zr without edge enrichment or isolated hot spots. This indicates that LiCl does not deposit as surface clusters but is distributed throughout the particle bulk. Note that EDS has inherently low sensitivity to Li; the absence of a Li map is a methodological limitation and does not affect the assignment based on Cl.

In the FTIR spectra (Figure 2d), UIO-66 displays coordinated carboxylate bands ν_as(COO^−^) ≈ 1580–1590 cm^−1^ and ν_s(COO^−^) ≈ 1395–1410 cm^−1^, while the PNIPAM hydrogel shows amide I ≈ 1650 cm^−1^ and amide II ≈ 1540–1550 cm^−1^. In the composite film, amide I/II undergo minor shifts (≈2–6 cm^−1^) with slight broadening, and the O–H stretching envelope (3200–3600 cm^−1^) becomes more intense. These changes point to hydrogen-bonding and ion–dipole interactions between PNIPAM amide groups and pore-confined LiCl/associated water, leading to more strongly bound water and broader bands; meanwhile, the carboxylate features of UIO-66 are largely retained, indicating that the framework coordination environment is unchanged. Such interfacial interactions are consistent with the amplified hygroscopic swelling and efficient strain transfer observed later in sensing tests, underpinning the high sensitivity.

Finally, the macroscopic pore structure of the hydrogel (Figure 2f) shows an ordered, honeycomb-like interconnected network in the dry state (scale bar 50 μm), with uniform walls and a relatively narrow pore-size distribution. This architecture provides low-resistance diffusion pathways and a large effective surface area, shortening the humidity-equilibration path and reducing mass-transport lag; combined with the annular geometry on the FBG, it enables efficient strain coupling—accounting for the faster response/recovery and higher wavelength sensitivity reported below.

### 3.2. Humidity Response as a Function of Coating Time and Optimal Coating Selection

**Figure 3:**
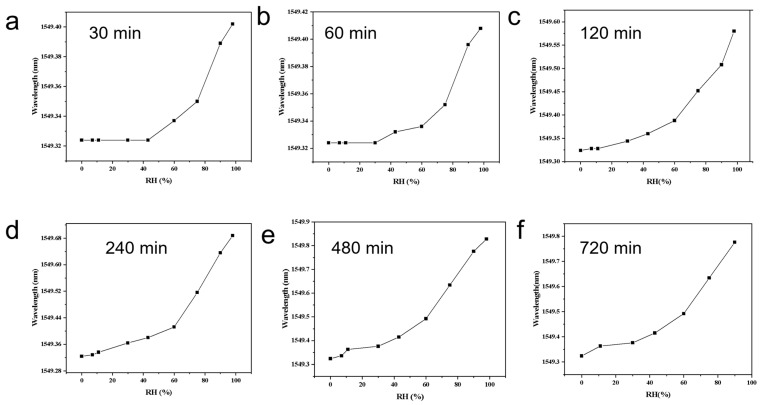
Bragg wavelength versus relative humidity (RH) for LiCl@UIO-66/PNIPAM-coated FBGs prepared with different coating times: (**a**) 30 min, (**b**) 60 min, (**c**) 120 min, (**d**) 240 min (4 h), (**e**) 480 min (8 h), and (**f**) 720 min (12 h). (The curves represent the measured response of individual sensors; therefore, error bars are not shown).

Under identical composition and test conditions, the hydrogel coating time on the FBG was varied while all other parameters were fixed. The Bragg wavelength versus relative humidity (RH) curves are presented individually as Figure 3a (30 min), Figure 3b (60 min), Figure 3c (120 min), Figure 3d (240 min, 4 h), Figure 3e (480 min, 8 h), and Figure 3f (720 min, 12 h). The total wavelength shift from ~0 to ~95% RH (Δλ) increases monotonically with coating time but with diminishing returns: ≈0.08 nm (Figure 3a), ≈0.09–0.10 nm (Figure 3b), ≈0.28–0.30 nm (Figure 3c), ≈0.38–0.40 nm (Figure 3d), ≈0.45–0.47 nm (Figure 3e), and ≈0.47–0.50 nm (Figure 3f). The corresponding average sensitivity (Δλ/ΔRH) therefore increases from ~0.8–1.0 pm/%RH for Figure 3a,b to ~5 pm/%RH for Figure 3e–f. Curve shape evolves: thin coatings (Figure 3a,b) are nearly flat at RH < 40% and show a marked upturn at RH > 60%, whereas thicker coatings (Figure 3c–f) show a lifted low-RH response and an overall quasi-linear profile with more pronounced high-RH gain.

To further understand the origin of this trend, the coating thickness was experimentally measured using optical microscopy on cross-sectioned fibers, yielding approximate values of ~5, 7, 12, 18, 22, and 24 μm for coating times of 30, 60, 120, 240, 480, and 720 min, respectively (Table S1). When correlated with the corresponding Δλ values (0.08–0.49 nm), the data clearly show that both layer thickness and sensitivity increase monotonically with coating time, but the growth rate progressively slows. The most substantial improvement occurs between 120 and 240 min (12 → 18 μm; Δλ 0.29 → 0.39 nm), whereas extending the coating time from 240 to 720 min results in only marginal gains (18 → 24 μm; Δλ 0.39 → 0.49 nm). This indicates that strain-transfer efficiency and hydration-induced swelling approach saturation at larger coating thicknesses due to diffusion limitations and relaxation constraints in the hydrogel–MOF–salt network.

These observations can be attributed to three principal mechanisms. First, volumetric sorption and strain amplification: thicker LiCl@UIO-66/PNIPAM layers provide larger sorption volume and longer diffusion pathways, enabling more complete hydration-induced swelling; the larger volumetric expansion produces greater axial tensile strain on the FBG and thus a larger Δλ. Second, strain-transfer efficiency increases with thickness: ultrathin coatings suffer from interface shear and radial confinement that limit axial strain transfer, while coatings that reach tens of micrometers approach a saturated transfer coefficient. Third, cooperative sorption and capillary condensation at high RH: PNIPAM osmotic pressure and capillary condensation within LiCl/UIO-66 micropores produce superlinear water uptake above ~60% RH, causing the high-RH upturn; thicker films with more active sites amplify this cooperative effect.

Optimal coating time: Considering the quantitative improvements in sensitivity, the experimentally measured thickness evolution, the diminishing returns beyond ~4 h, and the impact of film thickness on diffusion kinetics and mechanical reliability, a coating time of 4 h (≈18 μm) is identified as the optimal condition. At 4 h, Δλ ≈ 0.38–0.40 nm, corresponding to ~82% of the saturation value reached at 12 h. Extending coating time to 8–12 h increases Δλ by only ~0.06–0.09 nm (≈12–18% extra) while incurring penalties: slower response/recovery, larger hysteresis, and elevated risk of microcracking or delamination during cyclic operation. Therefore, 4 h provides an excellent balance between sensitivity, quasi-linearity, response speed, and mechanical robustness.

### 3.3. Humidity Response and Calibration of Different Materials

**Figure 4:**
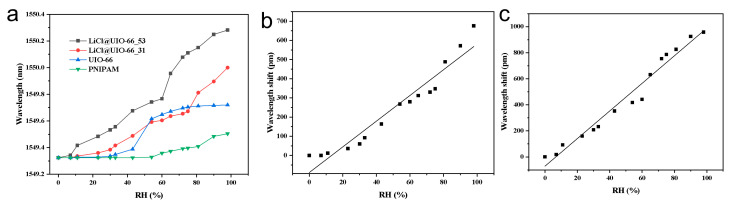
(**a**) Wavelength versus relative humidity for four coatings: PNIPAM, UIO-66, and LiCl@UIO-66/PNIPAM with lower and higher LiCl loadings; the higher-loading composite yields the largest shift, especially above ~60% RH. (**b**) Calibration curve of LiCl@UIO-66_33 with a sensitivity of 6.7 pm/%RH over ~0–95% RH. (**c**) Calibration curve of LiCl@UIO-66_51 with a sensitivity of 10.6 pm/%RH over the same range.

In Figure 4a, the wavelength responses of four coatings over a range of 0– ≈95% RH are compared: PNIPAM (matrix only), UIO-66 (without LiCl), and two LiCl@UIO-66/PNIPAM composites with low and high LiCl loadings. When RH < 30–40%, all curves are essentially flat which indicates limited water uptake and small axial strain at low humidity; when RH > 60%, the responses bend upward, with the high-LiCl composite showing the largest shift, followed by the low-LiCl composite, then UIO-66, and finally PNIPAM—the differences are most pronounced at ≈80–95% RH. This ordering is consistent with the structural characterization: the strong hygroscopicity of LiCl combined with micropore confinement in UIO-66 increases water content and volumetric swelling; ion–dipole/hydrogen-bonding interfacial coupling in the composite strengthens water binding; and the interconnected porous network together with a higher strain-transfer coefficient converts volume change more efficiently into axial strain on the FBG, producing a cooperative adsorption/capillary-condensation-driven upturn at high RH.

The corresponding linear calibrations are shown in Figure 4b,c. For LiCl@UIO-66_33 (Figure 4b), the response is approximately linear over a range of 0– ≈95% RH with a sensitivity of 6.7 pm/%RH; for LiCl@UIO-66_51 (Figure 4c), the linearity is even better and the sensitivity increases to 10.6 pm/%RH. Expressed in terms of wavelength shift Δλ (pm) versus RH (%), Δλ33 ≈ 6.7 × RH + b33, Δλ51 ≈ 10.6 × RH + b51, where the intercepts b_33_ and b_51_ are small and can be absorbed into zero-point calibration. The slope comparison shows that increasing the LiCl content raises the sensitivity from 6.7 pm/%RH to 10.6 pm/%RH. This improvement stems from stronger moisture capacity and pore-confined condensation leading to larger volumetric expansion, enhanced strain-transfer efficiency due to thicker coating and a more effective filler network, and PNIPAM–MOF–salt interfacial coupling that elevates hydration osmotic pressure, thereby converting changes in humidity more efficiently into Bragg wavelength shift.

To quantitatively verify the linearity and statistical significance of the humidity–wavelength relationship, we performed least-squares linear regression for the calibration curves of Figure 4b,c. In line with standard error-analysis practices used for evaluating the reliability of long-term correlated sensing and complex system modeling [42,43], the regression slope (sensitivity), coefficient of determination (R^2^), and statistical significance (*p* value) of the slope term were extracted. The results are summarized in Table 1.

As shown in Table S2, both composite coatings exhibit very high R^2^ values (0.969 and 0.976), confirming strong linearity across the tested RH range. The extremely small *p* values (4.4 × 10^−8^ and 6.3 × 10^−12^) indicate that the slopes are highly significant (*p* < 0.001), demonstrating that the observed wavelength shifts originate from real humidity-induced swelling rather than random noise. These results also statistically validate that increasing the LiCl loading leads to a physically meaningful enhancement in humidity sensitivity.

### 3.4. Response Kinetics Under Stepwise Humidity

**Figure 5:**
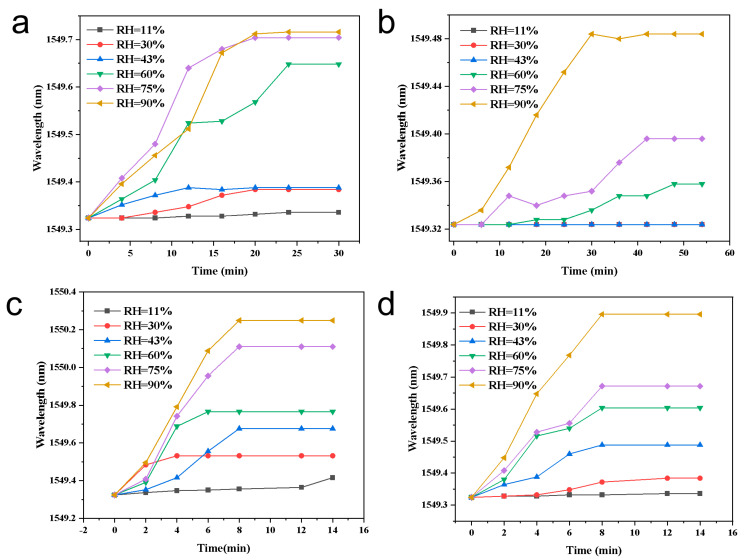
Time-resolved Bragg wavelength under stepwise RH (11%, 30%, 43%, 60%, 75%, and 90%) for different coatings: (**a**) pristine UIO-66; (**b**) PNIPAM without composite adsorbent; (**c**) LiCl@UIO-66/PNIPAM with 51 wt% LiCl; (**d**) LiCl@UIO-66/PNIPAM with 33 wt% LiCl.

Under stepwise humidity of RH = 11%, 30%, 43%, 60%, 75%, and 90%, we recorded the Bragg-wavelength–time traces for different coatings (Figure 5a–d) and defined the response time t90 as the time taken to reach 90% of the steady-state shift. The pristine UIO-66 coating (Figure 5a) exhibits a slow rise that levels off at about 30 min; the baseline PNIPAM coating without the composite adsorbent (Figure 5b) is even slower with t_90_ ≈ 55 min. In contrast, the two LiCl@UIO-66/PNIPAM composites—51 wt% LiCl (Figure 5c) and 33 wt% LiCl (Figure 5d)—both rise rapidly and settle within ≈14 min. Quantitatively, the composites compress t90t from ≈55 min (PNIPAM) to ≈14 min (3–4× faster) and from ≈30 min (UIO-66) to ≈14 min (~2× faster). Notably, the two loadings have a similar t_90_, but the 51 wt% sample delivers a larger displacement and higher sensitivity at high RH, consistent with the calibration results.

These kinetic differences are attributable to coupled mass-transport and interfacial effects. LiCl provides strong hygroscopicity and a high hydration number, creating a larger chemical-potential driving force for vapor ingress; UIO-66 contributes microporous/defect sites that enable rapid adsorption and capillary condensation, accelerating in-film uptake and homogenization. Within the PNIPAM network, the MOF–salt filler builds a percolated micro-pore/gel transport pathway and improves the strain-transfer coefficient to the FBG, turning volumetric swelling more efficiently into a Bragg wavelength shift. The near-equal t_90_ for 33 wt% and 51 wt% suggests that the rate-controlling step is dominated by in-film diffusion and polymer network relaxation, rather than simply by the number of active sites; increasing LiCl mainly boosts amplitude/sensitivity, not response time.

### 3.5. Long-Term Stability and Repeatability

**Figure 6:**
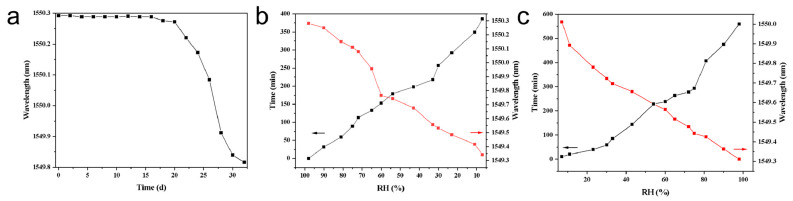
Long-term stability and repeatability of the hydrogel-coated FBG sensor based on LiCl@UIO-66/PNIPAM with 51 wt% LiCl. (**a**) Bragg wavelength remains essentially constant for the first ~20 days under constant conditions, followed by a slow decrease thereafter. (**b**,**c**) Repeatability upon stepwise decrease in RH from 100% to 10% using the same 51 wt% device: left axis—settling time; right axis—steady-state Bragg wavelength. The red and black lines in (**b**,**c**) represent Bragg wavelength and settling time, respectively, with arrows indicating key transition points.

Under constant ambient conditions, we monitored the hydrogel-coated FBG sensor fabricated with LiCl@UIO-66/PNIPAM containing 51 wt% LiCl (Figure 6a). The recorded Bragg wavelength remained essentially unchanged during the first 20 days, showing no drift beyond the instrument resolution; thereafter it exhibited a slow downward trend, indicating excellent zero-point stability in the short-to-mid term, with long-term drift emerging primarily after day 20. Figure 6b,c present repeatability/reversibility tests on the same 51 wt% device under a stepwise decrease in RH from 100% to 10%. The abscissa is RH; the left ordinate gives the settling time to steady state, and the right ordinate gives the corresponding steady-state Bragg wavelength. In both datasets, the steady-state wavelength decreases monotonically as RH decreases, while the settling time increases monotonically; the traces are smooth without anomalous jumps at each RH point, demonstrating good cycle-to-cycle consistency of the coated sensor.

These observations can be rationalized as follows. During humidity down-steps, the hydrogel dehydrates and shrinks, reducing the axial tensile strain on the FBG and therefore lowering the Bragg wavelength. As RH becomes lower, water in the film transitions from “free/weakly bound” to “strongly bound/hydration-layer”, so desorption kinetics and network relaxation become rate-controlling, which lengthens the settling time. For 51 wt% loading, pore-confined LiCl together with UIO-66 micropores increases water-binding strength and extends the desorption/diffusion path at low RH—this secures high sensitivity while also explaining the longer settling time toward the dry end. The slow drift after ~20 days in Figure 6a is plausibly associated with gradual water redistribution/structural relaxation within the film or trace water exchange with the environment.

## 4. Conclusions

A LiCl@UIO-66/PNIPAM composite hydrogel coating was integrated onto FBGs to realize high-sensitivity and wide-range humidity sensing. The MOF framework remained intact after LiCl loading, and the strong interfacial interactions within the composite enabled enhanced swelling and strain transfer, resulting in sensitivities of 6.7 and 10.6 pm/%RH and a response time of t_90_ ≈ 14 min. A 4 h coating time offered the best compromise between sensitivity, linearity, kinetics, and mechanical reliability, and the sensor demonstrated good short-term stability and repeatable behavior during humidity cycling.

Despite these advantages, the present work is limited by the absence of full adsorption–desorption hysteresis characterization, evaluation of long-term drift beyond ~30 days, and comprehensive temperature–humidity coupling or fatigue analyses. These aspects will be addressed in future studies through improved hysteresis mitigation, temperature compensation, and long-term reliability testing. Overall, the hydrogel–MOF–salt strategy provides a promising route to robust, EMI-immune FBG humidity sensors.

## Figures and Tables

**Table 1:** Relative humidity controlled by saturated salt solutions (25 °C).

Salt	RH (%) at 25 °C
LiBr	7
LiCl	11
CH_3_COOk	23
LiF	30
MgCl_2_	33
K_2_CO_3_	43
Mg(NO_3_)_2_ ①	54
NaBr	60
KI	65
NaNO_3_	72
NaCl	75
KBr	81
KNO_3_	90
Mg(NO_3_)_2_ ②	98

Notes: Mg(NO_3_)_2_ solution standing for 10 min yields ~54% RH; Mg(NO_3_)_2_ solution standing for 60 min yields ~98% RH at 25 °C. After changing the salt dish or solution, wait until the reference hygrometer reading stabilizes (≥20–30 min) before recording. To minimize history effects, perform measurements in cycles of increasing and then decreasing RH, and re-check the zero at the end of each cycle. ① Mg(NO_3_)_2_ solution standing for 10 min yields approximately 54% RH at 25 °C. ② Mg(NO_3_)_2_ solution standing for 60 min yields approximately 98% RH at 25 °C.

## Data Availability

The original contributions presented in this study are included in the article/Supplementary Material. Further inquiries can be directed to the corresponding authors.

## Supplementary Materials

The following supporting information can be downloaded at https://www.mdpi.com/article/10.3390/ma18245587/s1, Table S1. Coating time, film thickness, and wavelength shift Δλ. Table S2. Linear regression analysis of humidity calibration.
