# Supplementary material for: Development of a High-Sensitivity Humidity Sensor Using Fiber Bragg Grating Coated with LiCl@UIO-66-Doped Hydrogel
Source: Materials (Basel). 2025 Dec 12;18(24):5587. doi: 10.3390/ma18245587 (PMC12735255; doi:10.3390/ma18245587)
Supplement: Supplementary file 1 [file materials-18-05587-s001.zip › materials-3973563-supplementary.pdf]

Supplementary Information

Table S1. Coating time, film thickness, and wavelength shift Δλ.

Coating Time (min)	Thickness (μm)	Δλ (nm)
30	5	0.08
60	7	0.1
120	12	0.29
240	18	0.39
480	22	0.46
720	24	0.49

Table S1 summarizes the experimentally measured coating thickness and the corresponding Bragg wavelength shift (Δλ) for FBGs coated with LiCl@UIO-66/PNIPAM hydrogels at different deposition times.

Table S2. Linear regression analysis of humidity calibration.

Sample	Slope (pm/%RH)	R^2	p-value
LiCl@UIO-66_33	6.7	0.969	4.4×10^{-8}
LiCl@UIO-66_51	10.6	0.976	6.3×10^{-12}

Table S2 provides the regression slope (humidity sensitivity), coefficient of determination (R^2), and statistical significance (p value) obtained from least-squares fitting of the calibration curves shown in Figure 4b–c. These metrics quantitatively verify the linearity and reliability of humidity-induced wavelength shifts.
